# Active Immunization with an Octa-Valent *Staphylococcus aureus* Antigen Mixture in Models of *S*. *aureus* Bacteremia and Skin Infection in Mice

**DOI:** 10.1371/journal.pone.0116847

**Published:** 2015-02-24

**Authors:** Sanne van den Berg, Dennis G. A. M. Koedijk, Jaap Willem Back, Jolanda Neef, Annette Dreisbach, Jan Maarten van Dijl, Irma A. J. M. Bakker-Woudenberg, Girbe Buist

**Affiliations:** 1 Department of Medical Microbiology and Infectious Diseases, Erasmus University Medical Center, Rotterdam, the Netherlands; 2 Department of Medical Microbiology, University of Groningen, University Medical Center Groningen, Groningen, the Netherlands; 3 Pepscan Therapeutics BV, Lelystad, the Netherlands; Instituto Butantan, BRAZIL

## Abstract

Proteomic studies with different *Staphylococcus aureus* isolates have shown that the cell surface-exposed and secreted proteins IsaA, LytM, Nuc, the propeptide of Atl (pro-Atl) and four phenol-soluble modulins α (PSMα) are invariantly produced by this pathogen. Therefore the present study was aimed at investigating whether these proteins can be used for active immunization against *S*. *aureus* infection in mouse models of bacteremia and skin infection. To this end, recombinant His-tagged fusions of IsaA, LytM, Nuc and pro-Atl were isolated from *Lactococcus lactis* or *Escherichia coli*, while the PSMα1-4 peptides were chemically synthesized. Importantly, patients colonized by *S*. *aureus* showed significant immunoglobulin G (IgG) responses against all eight antigens. BALB/cBYJ mice were immunized subcutaneously with a mixture of the antigens at day one (5 μg each), and boosted twice (25 μg of each antigen) with 28 days interval. This resulted in high IgG responses against all antigens although the response against pro-Atl was around one log lower compared to the other antigens. Compared to placebo-immunized mice, immunization with the octa-valent antigen mixture did not reduce the *S*. *aureus* isolate P load in blood, lungs, spleen, liver, and kidneys in a bacteremia model in which the animals were challenged for 14 days with a primary load of 3 × 10^5^ CFU. Discomfort scores and animal survival rates over 14 days did not differ between immunized mice and placebo-immunized mice upon bacteremia with *S*. *aureus* USA300 (6 × 10^5^ CFU). In addition, this immunization did not reduce the *S*. *aureus* isolate P load in mice with skin infection. These results show that the target antigens are immunogenic in both humans and mice, but in the used animal models do not result in protection against *S*. *aureus* infection.

## Introduction

*Staphylococcus aureus* is a widespread Gram-positive bacterium that colonizes the skin and anterior nares of about 20–30% of the healthy human population [1]. Although mainly a harmless colonizer, *S*. *aureus* can cause invasive diseases like skin and soft tissue infections, and can be responsible for severe infections in humans like pneumonia, endocarditis and osteomyelitis [1], which are frequently associated with *S*. *aureus* bacteremia [2]. *S*. *aureus* in its methicillin-resistant form (MRSA) is the most important cause of antibiotic-resistant health care-associated infections worldwide [3,4]. In the case of MRSA, a single genetic element makes *S*. *aureus* resistant to the most frequently prescribed class of antimicrobials—the β-lactam antibiotics, including penicillins, cephalosporins, and carbapenems [5]. A high incidence of MRSA is encountered in hospitals, resulting in prolonged hospital stays and in higher mortality rates [3,4], and limited effectiveness of alternative treatment regimens. Glycopeptides, especially vancomycin, are currently used as first-line treatment of MRSA infections. Unfortunately, this has led to the emergence of vancomycin-intermediate and vancomycin-resistant MRSA [6]. In addition, there is raising concern that the current first-line treatment for MRSA infection will become increasingly ineffective. Since in the past 25 years no novel small-molecule antibacterial drugs have been discovered [7], and the development pipeline of new antimicrobials remains lean [8], new ways of treatment of *S*. *aureus* infections such as immunization need to be explored. Several strategies of passive and active immunization in *S*. *aureus* infections have been studied in experimental infection models, but until now none of these have been proved to be effective in clinical studies [9–11]. Insufficient power because of a low sample size in some clinical studies [12,13], as well as the heterogeneity of *S*. *aureus* strains causing infections in humans [14,15] may contribute to the failure of treatment through immunization in patients. Despite the lack of success so far, immunization approaches are still worth pursuing especially in patients admitted to the hospital for elective surgery. For this group of patients, there may be enough time for immunization prior to surgery.

Novel target identification strategies have been applied to screen for new antigenic targets for immunization. Invariant immunogenic determinants of relevant *S*. *aureus* isolates have been successfully identified in previous studies using a combination of proteomics, genomics, bioinformatics and immunological approaches [15–18]. A complete inventory of predicted secreted proteins of sequenced *S*. *aureus* strains has been made [16]. Proteomic analysis of the exoproteomes of 25 clinical *S*. *aureus* isolates showed that only seven of these secreted proteins (IsaA, Lip, LytM, Nuc, SA0620, SA2097, and SA2437) were produced by all clinical isolates studied [15]. In a proteolytic shaving approach of *S*. *aureus* cells, multiple surface-exposed proteins were identified among which IsaA, Nuc, Atl and the phenol-soluble modulin (PSM) α1 peptide [17].

In the present study, we used IsaA, LytM, Nuc, pro-Atl, and the PSMα1-4 peptides as targets for active immunization in *S*. *aureus*-infected mice. These *S*. *aureus* antigens were all selected by the previous target identification strategies, and are all potential virulence factors of *S*. *aureus*. The PSMs are highly potent surfactants that facilitate the movement of *S*. *aureus* over moist surfaces by colony spreading, and they are involved in the metastatic escape of staphylococcal cells from biofilms thereby representing an enormous risk factor for serious invasive disease [19,20]. High levels of PSM production are supposed to contribute to the high virulence and epidemic behavior of community-acquired MRSA lineages [21].

Staphylococcal major autolysin (Atl) is a bifunctional autolysin composed of a signal peptide for protein secretion, a propeptide, and domains with amidase and glucosaminidase activity. After proteolytic cleavage, the active domains are involved in cell separation and they have been shown to be cell surface-exposed [22]. It is believed that the propeptide of Atl (pro-Atl) has a role in the folding and/or activation of Atl and that it would be degraded rapidly upon proteolytic processing of the Atl protein.

Immunodominant staphylococcal antigen A (IsaA) and autolysin M (LytM) have been shown to be peptidoglycan hydrolases with transglycosylase and glycyl-glycine endopeptidase specific activity, respectively [18]. The secreted nuclease (Nuc) has been shown to limit the formation of biofilms due to the degradation of extracellular DNA [23]. This protein also promotes resistance against neutrophil extracellular traps-mediated antimicrobial activity of neutrophils and thus contributes to disease pathogenesis *in vivo* [24].

In previous studies, IsaA, LytM, Nuc, pro-Atl and PSMα1-4 have all been shown to be immunogenic. Using a multiplex assay to quantify antibody responses against 26 staphylococcal proteins, we found that mice with a *S*. *aureus* USA300 pneumonia or skin infection showed good IgG responses against IsaA and Nuc, while anti-LytM levels were low [25]. In contrast, in mice immunized with monovalent staphylococcal vaccines containing IsaA, Nuc or LytM, the highest IgG responses were obtained against the latter antigen. Using the same multiplex assay, it has been shown that 75% of the patients with the genetic blistering disease epidermolysis bullosa (EB), who were heavily colonized with multiple *S*. *aureus* types, have increased IgG responses against some secreted, cell wall and membrane-bound staphylococcal proteins [26,27]. Importantly, EB patients do not frequently suffer from *S*. *aureus* bacteremia, which suggests that their high anti-staphylococcal antibody titers might be protective against invasive *S*. *aureus* infections. Within the EB patient group, the highest response was detected against IsaA, while for Nuc and LytM moderate responses were detected. These data show that both in mice and in human individuals a good IgG response is generated against IsaA, LytM and Nuc. Moreover, passive immunization of mice with murine anti-IsaA resulted in protection against staphylococcal infection both in a central venous catheter-related infection model and a sepsis survival model, most likely due to phagocytosis that resulted in killing of *S*. *aureus* [28]. Holtfreter et al. showed in their review that Atl has been identified in different studies as a well-recognized target of the human immune system [29]. Recently, we showed that pro-Atl is exposed on the outside of *S*. *aureus* cells and is recognized by antibodies from four out of six patients that were colonized with *S*. *aureus* (our unpublished observations). Within a patient group of hospitalized adults with an invasive *S*. *aureus* infection, in those patients who did not develop sepsis, higher levels of immunoglobulin G (IgG) responses were detected against exotoxins among which PSMα3 [30].

In the present study in mice, we examined the protective efficacy of active immunization with an octa-valent antigen mixture containing IsaA-His_6_, LytM-His_6_, Nuc-His_6_, His_6_-pro-Atl and the PMSα1–4 peptides. In mice immunized with a mixture of these antigens, the generated IgG response was determined by enzyme-linked immunosorbent assays (ELISA). Subsequently, the protective capacity of this immunization strategy was studied in mouse models of methicillin-sensitive *S*. *aureus* (MSSA) or MRSA bacteremia and in a mouse model of MSSA skin infection, which are clinically highly relevant *S*. *aureus* infections [31].

## Materials and Methods

### Bacterial strains and growth conditions

Strains and plasmids used in this study are listed in Table 1. *Lactococcus lactis* strains were grown at 30°C in M17 broth (Oxoid Ltd, Hampshire, UK) supplemented with 0.5% w/v glucose (GM17). When necessary the medium was supplemented with chloramphenicol (5 μg/mL) for plasmid selection. For *in vivo* studies, *S*. *aureus* isolate P and USA300 were used and grown in Brain Heart Infusion broth (Becton Dickinson, Breda, the Netherlands). *S*. *aureus* isolate P is a community-acquired MSSA strain recovered from the blood of a septic patient and was previously analyzed by proteomics [15]. *S*. *aureus* USA300 is one of the most frequent causes of community-acquired infections in the United States of America [32].

**Table 1:** Bacterial strains and plasmids used in this study.

Strain or plasmid	Relevant phenotype(s) or genotype(s)	Source or reference
**Strains**		
*E*. *coli* BL21DE3	Allows IPTG-inducible expression of P_T7_	Novagen
*L*. *lactis* NZ9700	Nisin producer	[63]
*L*. *lactis* PA1001	MG1363 *pepN*::*nisRK*, allows nisin inducible expression, Δ*acmA* Δ*htrA*	[64]
*S*. *aureus* isolate P	Community-acquired MSSA patient isolate	[15]
*S*. *aureus* USA300	Community-acquired MRSA isolate	[32]
*S*. *aureus* N315	Hospital-acquired MRSA	[65]
**Plasmids**		
pET24d::*isaA*::*his*_*6*_	*Kan* ^*R*^, pET24d containing *isaA* with C-terminal *his*_*6*_	[34]
pPA180::*lytM*::*his*_*6*_	*Cm* ^*R*^, pPA180 containing *lytM* with C-terminal *his*_*6*_	[25]
pNG400::*nuc*::*his*_*6*_	*Cm* ^*R*^, nisin inducible expression via P_*nisA*_ of Nuc with C-terminal *his*_*6*_	[34]
pNG4110::*proAtl*	*Cm* ^*R*^, nisin inducible expression via P_*nisA*_ of the pro-Atl peptide fused to SS_*usp45*_, and a N-terminal *his*_*6*_	This study

*Kan* ^*R*^, kanamycin resistance gene; *Cm* ^*R*^, chloramphenicol resistance gene; P_T7_, IPTG inducible T7-promoter; P_*nisA*_, nisin inducible promoter; *his*_*6*_, 6 histidine-tag; SS_*usp45*_, signal sequence of *usp45*.

### Construction of His_6_-pro-Atl expression plasmids

For cloning of the DNA fragment coding for the propeptide of Atl (pro-Atl) in the plasmid pNG4110, the primers atlpro.F1 (ATATGGATCCGCTGAGACGACACAAGATCAAACTACTAATAAAAACG) and atlpro.R2 (ATATGCGGCCGCTTAAGCGCTAAAAGTAGTTACTTTAGGTGTCGCTTCAGTTTTAGC) were used with chromosomal DNA of *S*. *aureus* strain N315 as a template. Using this vector, a N-terminally His-tagged fusion protein can be secreted from *L*. *lactis* [33]. PCR product and vector were digested using *Bam*HI and *Not*I (cleavage sites are underlined in the primers sequences). After ligation the resulting plasmids were transferred into *L*. *lactis* PA1001 by electro transformation with selection on chloramphenicol. A nucleotide sequence analysis of the cloned inserts was performed by Eurofins DNA (Ebersberg, Germany).

### Protein isolation, purification and quantification

For the production and isolation of IsaA-His_6_, an overnight culture of *E*. *coli* BL21DE3 (pET24d::*isaA*::*his*_*6*_) was diluted 1:100 in fresh lysogeny broth with 50 μg/mL kanamycin [34]. Induction was performed at OD_600_ ~ 0.5 for 4 h by adding 1 mM isopropyl β-D-1-thiogalactopyranoside (IPTG) (Duchefa, Haarlem, the Netherlands). The culture was centrifuged and the pellet was resolved in binding buffer (20 mM Na-Phosphate, pH 7.4, 0.5 M NaCl_2_, 60 mM imidazole) containing 6 M urea. After sonification (Sonicator S-4000, Misonix, Farmingdale, USA) the supernatant was mixed with binding buffer (1:1) and HisLink beads (Promega, Madison, USA) for 1 h at 4°C under shaking conditions. Protein elution was performed with binding buffer containing 500 mM imidazole.

For the production and isolation of LytM-His_6_ [25], Nuc-His_6_ [25] and His_6_-pro-Atl, overnight cultures of *L*. *lactis* PA1001 were diluted 1:2 in GM17 medium. Nisin induction for protein expression was performed at OD_600_ ~ 0.5 for 16 h by adding the culture supernatant (1:1000) from an overnight culture of *L*. *lactis* NZ97000. For analysis of the extracellular production of His_6_-pro-Atl by *L*. *lactis* PA1001 (pNG4110::*proAtl*), proteins from the culture supernatant were precipitated using trichloroacetic acid (TCA; 10% w/v). The culture was centrifuged and the pellet was washed with acetone and dried. For the isolation of Nuc-His_6,_ from the supernatant fraction of a culture of *L*. *lactis* PA1001 (pNG400::*nuc*::*his*_*6*_), HisLink beads were added for 1 h at 4°C under shaking conditions after which HisLink beads were collected. For isolation of LytM-His_6_ cells of *L*. *lactis* PA1001 (pPA180::*lytM*::*his*_*6*_) were harvested and disrupted in binding buffer using a Sonicator S-4000. Purification was performed with Mag beads (GE Healthcare, Uppsala, Sweden) for 1.5 h under shaking conditions at 4°C. Elution of Nuc-His_6_ and LytM-His_6_ was done with binding buffer containing 500 mM imidazole. The flow-through, wash and elution fractions were analyzed by LDS-PAGE. Protein samples were mixed with LDS buffer and incubated at 95°C for 10 min, separated by LDS-PAGE using precast 10% NuPage gels (Life Technologies, Bleiswijk, the Netherlands) and stained with Simply Blue™ Safe Stain (Life Technologies).

The fractions containing the purified His-tagged proteins of IsaA-His_6_, LytM-His_6_, Nuc-His_6_ or His_6_-pro-Atl were pooled and dialyzed (G2-Float-A-Lyzer, Spectrum Europe BV, Breda, the Netherlands) against PBS and concentrated with the Speedvac Concentrator Plus (Eppendorf Nederland BV, Nijmegen, the Netherlands).

Protein concentrations were determined with the DC Protein Assay (Bio-Rad, Veenendaal, the Netherlands) according to the instructions of the supplier, using Bovine Serum Albumin (Sigma-Aldrich, Zwijndrecht, the Netherlands) as a standard, or with the Nanodrop ND-1000 (Thermo Fisher Scientific, Wilmington, Delaware USA) using the extinction coefficient calculated for each of the proteins.

PSMα1-4 were synthesized as described previously [19], with the addition of a GGG-Lys(ε-biotin). The peptides were mixed in a 1:1:1:1 molar ratio, and incubated in PBS in a stoichiometric ratio to avidin (Thermo Fisher Scientific Inc., Rockford, USA).

### Human plasma

Whole blood donations from EB patients were collected under the approval of the medical ethics committee of the University Medical Center Groningen (approval no. NL27471,042,09) upon written informed patient consent, and with adherence to the Helsinki Guidelines [26]. The Independent Ethics Committee of the Foundation ‘Evaluation of Ethics in Biomedical Research’ (Assen, the Netherlands), approved the protocol for blood donations from healthy volunteers. This protocol is registered by QPS Groningen (code 04132-CS011). The required written informed consent was obtained from all EB patients and healthy volunteers included in the present studies.

### Protein detection and activity assays

For Western blot analyses, proteins separated by LDS-PAGE were blotted onto a nitrocellulose membrane (Protran, Schleicher & Schuell BioScience, Dassel, Germany). Immunodetection was performed using anti-His-tag antibodies (Invitrogen, Life Technologies) and rabbit polyclonal antibodies raised against the amidase or glucosaminidase domains of Atl (gift from Motoyuki Sugai, Hiroshima University, Japan [35]). Dilutions (1:1000) of the collected human plasma [26] were used to detect the IgG responses against IsaA-His_6_, LytM-His_6_ and Nuc-His_6_. Equal amounts of the three proteins were loaded on the gel and after blotting strips of the blot were incubated with the different plasma samples. Bound primary antibodies were visualized using specific fluorescently labeled secondary antibodies (IRDye 800 CW, Li-Cor Biosciences, Lincoln, USA). Membranes were scanned for fluorescence at 800 nm using the Odyssey Infrared Imaging System (Li-Cor Biosciences, Nebraska, USA).

To show expression of the PSMα1-4 peptides by *S*. *aureus* isolates P and USA300, both strains were tested for the ability to spread on tryptic soy soft agar (TSA) plates (0.24% agar). From an overnight culture, an aliquot of 2 μL was spotted in the middle of a TSA plate, which was then incubated overnight at 37°C. The spreading assay was performed as described [19,36].

Peptidoglycan hydrolase activity of IsaA-His_6_ and LytM-His_6_ was detected by a zymogram staining technique using SDS-polyacrylamide (12.5%) gels containing 0.1% (w/v) autoclaved cell wall fragments of *S*. *aureus* RN4220 isolated as described previously [37]. After electrophoresis, the gels were gently shaken at room temperature for 24 h in three to five changes of 100 mL of 25 mM Tris-HCl (pH 6.0) containing 1% (v/v) Triton X-100 for protein renaturation.

### Pepscan analysis

To determine whether regions of the *S*. *aureus* PSMα1-4 peptides were recognized by human IgGs, libraries of linear 15-mer peptides were synthesized with an overlap on solid support (Pepscan), as previously described [38]. The peptide libraries were probed with heat-inactivated human sera, in a dilution of 1:1000, with goat-anti-human-HRP conjugate (SouthernBiotech, Birmingham, USA) as a secondary antibody, and developed with 2,2'-azino-bis(3-ethylbenzothiazoline-6-sulphonic acid (Sigma-Aldrich). A charge-coupled device camera was used to register absorbance at 405 nm. For every single Pepscan dataset, the data were normalized to the average signal intensity of the analysis. Furthermore, the signals for every single protein were normalized to the median of the corresponding protein. In addition the standard deviations of the normalized data sets were calculated for each protein. Peptides with a signal exceeding the median plus twice the standard deviation and normalized signal intensity higher than three were regarded as being immunogenic domains.

### Animals

Specified pathogen-free female BALB/cBYJ mice were obtained from Charles River (Saint-Germain-sur-l’Arbresle, France). Mice were housed in individually ventilated cages, 3–4 mice per cage. Animals were 11–13 weeks old at the day of infection, and were given food and water ad libitum. All animal experiments were performed in accordance with the rules laid down in the Dutch Animal Experimentation Act and the EU Animal Directive 2010/63/EU (permit number: EMC2694).

### Immunization procedure

Purified Nuc-His_6_, LytM-His_6_, IsaA-His_6_, His_6_-pro-Atl, and PSMα1-4 were emulsified 1:1 with TiterMax Gold adjuvant (Sigma-Aldrich). Mice were immunized subcutaneously in the flank with 100 μL formulated vaccine on days -70 (5 μg of each antigen), days -42, and -14 (25 μg of each antigen). PSMα1-4 was considered as a single antigen, and therefore a total of 5 or 25 μg of this 1:1:1:1 mixture was used per immunization. Control mice received 100 μL PBS emulsified with adjuvant. Mice were randomly allocated to either the vaccine or the placebo group. At days -71 and -1, blood was withdrawn from the tail artery. Sera were examined by ELISA for IgG titers with specific antigen-binding activity.

### ELISA

ELISA plates (Greiner Bio-One B.V, Alphen aan den Rijn, the Netherlands) were coated with 250 ng of the antigens in coating buffer (0.05 M carbonate-bicarbonate, pH 9.6–9.8) and incubated for three days at 4°C. The plates were blocked for 45 min at 37°C in coating buffer with 2.5% milk powder (Oxoid, Hampshire, UK). After washing, serial twofold dilutions of the sera were made in PBS containing 0.05% Tween-20 (PBST) and incubated for 2 h at 37°C. After washing with PBST, the plates were incubated with GaM/IgG-HRP (SouthernBiotech) (1:5000 in PBST) for 90 min at 37°C. Finally, the peroxidase reaction was visualized using o-phenylene-diamine (Sigma-Aldrich) for 30 min at room temperature. The reaction was stopped by adding 2 M H_2_SO_4_. The plates were measured at 492 nm in a plate reader (Biotek Powerwave XS2, Beun de Ronde, Abcoude, the Netherlands).

### Infection model of *S*. *aureus* bacteremia

Immunized mice (n = 8 per group) were challenged on day 0 by intravenous inoculation of 100 μL of *S*. *aureus* isolate P (3 × 10^5^ CFU) or *S*. *aureus* USA300 (6 × 10^5^ CFU) as described previously [39]. Discomfort and animal survival rate over 14 days after infection were monitored. For discomfort score, clinical signs of illness in each mouse were evaluated at least twice daily as described before [39]. Mice were scored -1 directly after bacterial inoculation. Mice with bad fur were scored -2. Mice with bad fur and hunched back were scored -3. Mice with bad fur and hunched back and that were instable were scored -4. These mice showed severe signs of illness and were euthanized by CO_2_ exposure. At day 14 after infection in surviving mice, *S*. *aureus* load in blood, lungs, spleen, liver and kidneys was determined. Mice were sacrificed by CO_2_ exposure and exsanguinated by cardiac puncture. Blood was collected in a vial containing Lithium Heparin (Sarstedt, Etten-Leur, the Netherlands). Organs were removed aseptically and homogenized using a gentleMACS™ Dissociator (Miltenyi Biotec, Leiden, the Netherlands) in 2 mL of saline. CFUs of (un-)diluted blood and organ homogenates were determined after overnight growth on trypticase soy agar with 5% sheep blood (Becton Dickinson).

### Infection model of *S*. *aureus* skin infection

Immunized mice (n = 4 per group) were challenged on day 0 by intradermal inoculation of *S*. *aureus* isolate P. The method of induction of *S*. *aureus* skin infection in mice was adapted from Brown et al. [40]. In short, the lower back of the mice was shaved and cleaned with 70% ethanol under general anesthesia after using a mixture of medetomidine (Sedator^®^, 0.5 mg/kg; Eurovet Animal Health, Bladel, the Netherlands), midazolam (Midazolam, 5 mg/kg; Actavis, Baarn, the Netherlands) and fentanyl (Fentanyl, 0.05 mg/kg; Hameln Pharmaceuticals, Hameln, Germany). *S*. *aureus* isolate P (3 × 10^7^ CFU) was injected intradermally (50 μL). The mice were antagonized using a mixture of atipamezole (Antisedan^®^, 2.5 mg/kg; Orion Corporation, Espoo, Finland), flumazenil (Flumazenil, 0.5 mg/kg; Pharmachemie, Haarlem, the Netherlands) and naloxon (Naloxon, 1.2 mg/kg; Orpha-Devel Handels und Vertriebs, Purkersdorf, Germany). Anesthetic and antagonistic agents were administered intraperitoneally, in a total volume of 175 and 250 μL, respectively. Animal body weight and lesion size over 7 days after infection were monitored. Lesions were measured with a caliper. Lesion size was calculated by using the formula *A* = π(*L* × *W*)/2, where *L* is length and *W* is width of the lesion [41]. At day 7 after infection, mice were sacrificed by CO_2_ exposure. A circular area (diameter 14 mm) of the skin lesion was removed aseptically and homogenized using a gentleMACT™ Dissociator in 2 mL saline. Serial dilutions of skin homogenates were cultured on phenol-red mannitol salt agar (Becton Dickinson). Culture plates were incubated at 35°C for 48 h and at room temperature for 5 days. Identification of *S*. *aureus* was based upon colony morphology on the PHMA. Suspected colonies were cultured overnight on trypticase soy agar with 5% sheep blood (Becton Dickinson). A latex agglutination test (Staph Plus Latex; DiaMondial, Vienna, Austria) was then performed. The primary outcome measure was a reduction in *S*. *aureus* load in the skin lesion, secondary outcome measures were animal body weight and skin lesion size over time.

### Statistical analysis

The Mann-Whitney U test was used to compare median differences in bacterial load in different groups. The log rank test was used to determine statistical differences in animal survival rate between groups. Correlations between IgG titers and time-to-death were assessed using Pearson’s correlation coefficient. GraphPad Prism 5 for Windows (GraphPad Software Inc., La Jolla, CA) was used for these statistical analyses. Quade’s rank analysis of covariance was used to compare discomfort score, skin lesion size and body weight in different groups over time. These statistical analyses were performed using the Statistical Package of Social Sciences version 17.0 for Windows (SPSS Inc., Chicago, IL). *P*-values < 0.05 were considered to be statistically significant.

## Results

### Isolation and purification of *S*. *aureus* antigens

In a recent study on epitope mapping of surface proteins of *S*. *aureus*, the N-terminal propeptide of Atl (pro-Atl) was shown to be cell surface exposed (our unpublished observations). Therefore, this protein domain was chosen as a candidate target for the screening of human antibodies against *S*. *aureus*. After cloning, the His_6_-pro-Atl fusion protein was produced by *L*. *lactis* and expression and secretion of the peptide was proven by comparing the cell and growth medium fractions of nisin-induced and non-induced cultures using LDS-PAGE analysis as previously described [34] (data not shown). Western detection confirmed that this recombinant protein contained the His-tag needed for its isolation (data not shown).

**Fig 1:**
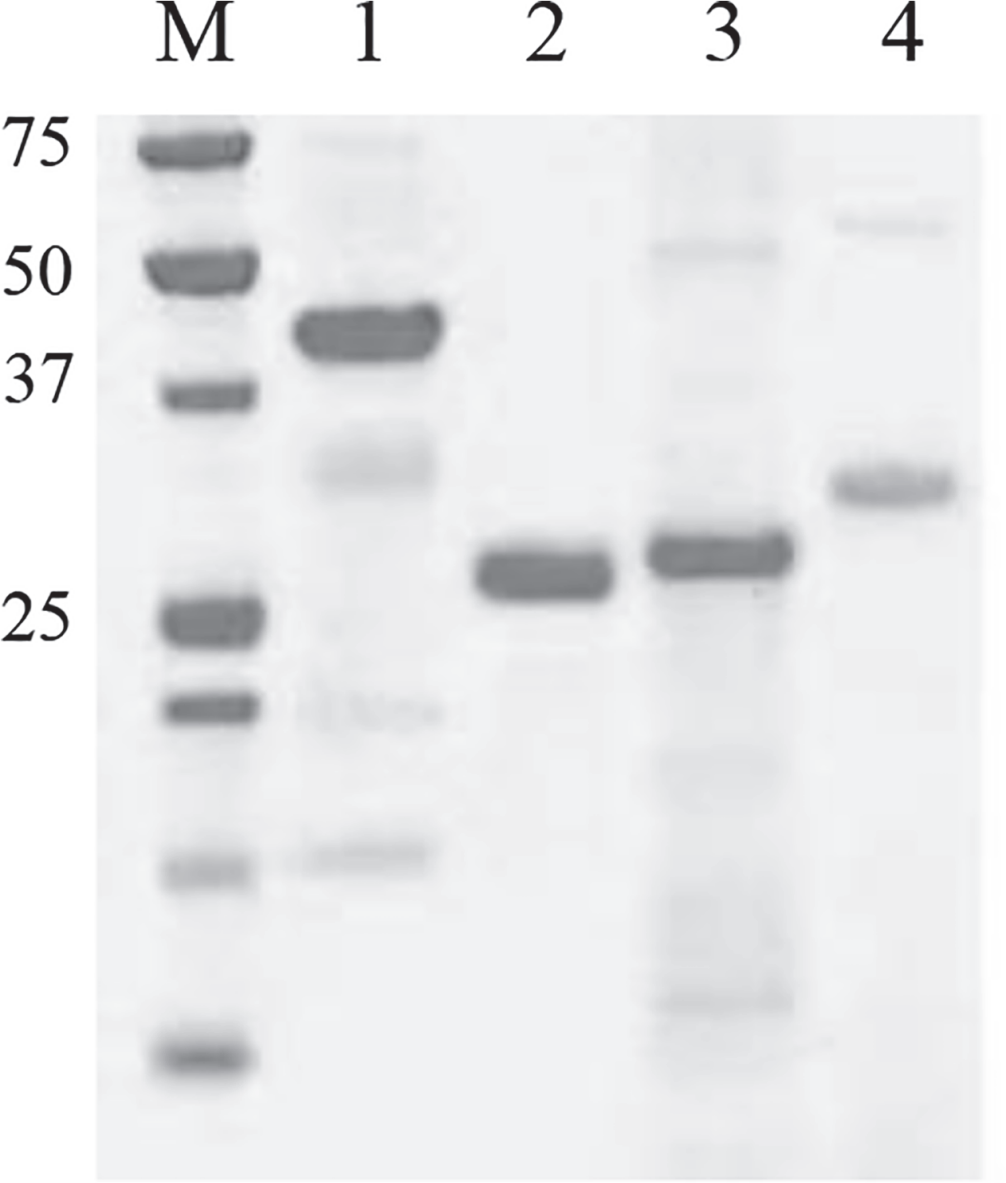
LDS-PAGE detection of the purified and dialyzed *S*. *aureus* antigens. Fifteen μg of LytM-His6 (1), Nuc-His6 (2), IsaA-His6 (3) or His6-pro-Atl (4) was loaded. The molecular weight of marker proteins is indicated (in kDa).

The *S*. *aureus* antigen IsaA-His_6_ was isolated from *E*. *coli*, whereas LytM-His_6_, Nuc-His_6_, and His_6_-pro-Atl were isolated from *L*. *lactis* to avoid possible cloning, degradation and expression problems [34]. The isolated and purified His-tagged proteins were verified using LDS-PAGE and subsequent protein staining (Fig. 1). Using zymographic analysis it was shown that purified LytM-His_6_ and IsaA-His_6_ had retained their enzymatic peptidoglycan-degrading activity while Nuc-His_6_ was still able to hydrolyze DNA [34]. The individual and pooled synthetic PSMα1-4 peptides were shown to facilitate the spreading motility of a non-motile *S*. *aureus* strain that cannot produce these peptides [36], indicating that they are biologically active [19]. These data show that the antigens were successfully isolated or synthesized and that the IsaA-His_6_, LytM-His_6_, and Nuc-His_6_ fusions had retained their enzymatic activities.

### Production of antigens by S. aureus isolate P and USA300

To show the production of the selected antigens by the *S*. *aureus* isolates P (MSSA) and USA300 (MRSA) that were used in the *in vivo* studies, different protein detection methods were used. For *S*. *aureus* USA300 all antigens used in this study had been identified in earlier (proteomics) studies (Table 2). Also for the *S*. *aureus* isolate P, most of the antigens were identified by proteomics in earlier studies (Table 2). Expression of the Atl protein by *S*. *aureus* isolate P was detected using specific polyclonal antibodies as mentioned in the Materials and Methods (data not shown). As pro-Atl is the propeptide of the autolysin Atl, which was detected by proteomic studies, pro-Atl was concluded to be expressed by both *S*. *aureus* strains (our unpublished observations). The expression of the PSMα1-4 peptides has been detected indirectly by using a plate assay in which cells of *S*. *aureus* isolates P or USA300 showed a spreading phenotype on a soft agar plate, indicating that both strains produce the PSM peptides (Table 2, data not shown). These data show that all antigens used in this study are produced and secreted by *S*. *aureus* isolates P and USA300.

**Table 2:** Proteins and peptides used in this study.

Name	NCBI number / sequence*	Function	Identification antigen in		Production	Mw (dD)	PI
			isolate P	USA300			
IsaA-His_6_	SA2356 (N315)	Transglycosylase	(1)	(2)	*E*. *coli*	22.7	6.3
Nuc-His_6_	SA0746 (N315)	Thermonuclease	(1)	(3)	*L*. *lactis*	20.1	9.7
LytM-His_6_	SA0265 (N315)	Glycyl-glycine endopeptidase	(1)	(3)	*L*. *lactis*	33.1	6.4
His_6_-pro-Atl	USA300HOU_0997 (USA300)	Propeptide autolysin Atl	Western (5)	(1)	*L*. *lactis*	19.4	7.9
PSMα1	MGIIAGIIKVIKSLIEQFTGKGGGGK$#	Spreading/Toxin	Spreading (6)	(2)/(4)	Synthetic	3.07	10.5
PSMα2	MGIIAGIIKFIKGLIEKFTGKGGGGK$#	Spreading/Toxin	Spreading (6)	(2)/(4)	Synthetic	3.09	10.7
PSMα3	MEFVAKLFKFFKDLLGKFLGNNGGGGK$#	Spreading/Toxin	Spreading (6)	(2)/(4)	Synthetic	3.41	10.4
PSMα4	MAIVGTIIKIIKAIIDIFAKGGGGK$#	Spreading/Toxin	Spreading (6)	(2)/(4)	Synthetic	2.98	10.6

* the sequences GGK$# stand for the addition of the GGG-Lys(ε-biotin) to each of the peptides

(1) Identified by proteomics [15]

(2) Identified by proteomics [17]

(3) Identified by proteomics (our unpublished observations)

(4) Identified using spreading assay [19]

(5) detection of Atl using specific antibodies (see Materials and Methods)

(6) Spreading as determined by plate assay (results not shown)

### Detection of IgG responses against the selected antigens in EB patients

**Fig 2:**
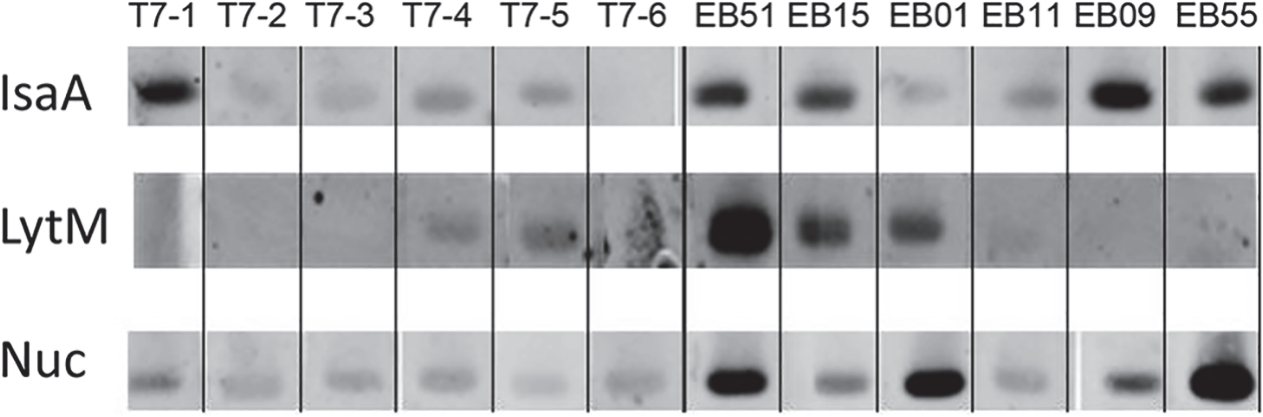
Western detection of immune responses against the antigens IsaA-His_6_, LytM-His_6_ and Nuc-His_6_. Sera of healthy volunteers (T7-1 till T7-6) and EB patients (EB51, 15, 01, 11, 09, 55) were used.

To assess the IgG responses against IsaA-His_6_, LytM-His_6_ and Nuc-His_6,_ immunodetection was performed using sera of healthy donors and EB patients (Fig. 2). From this analysis it is clear that EB patients have, on average, a higher although variable IgG response against all three proteins in comparison to the healthy donors. The Atl propeptide was shown to be recognized by antibodies present in sera of EB patients (our unpublished observations).

**Fig 3:**
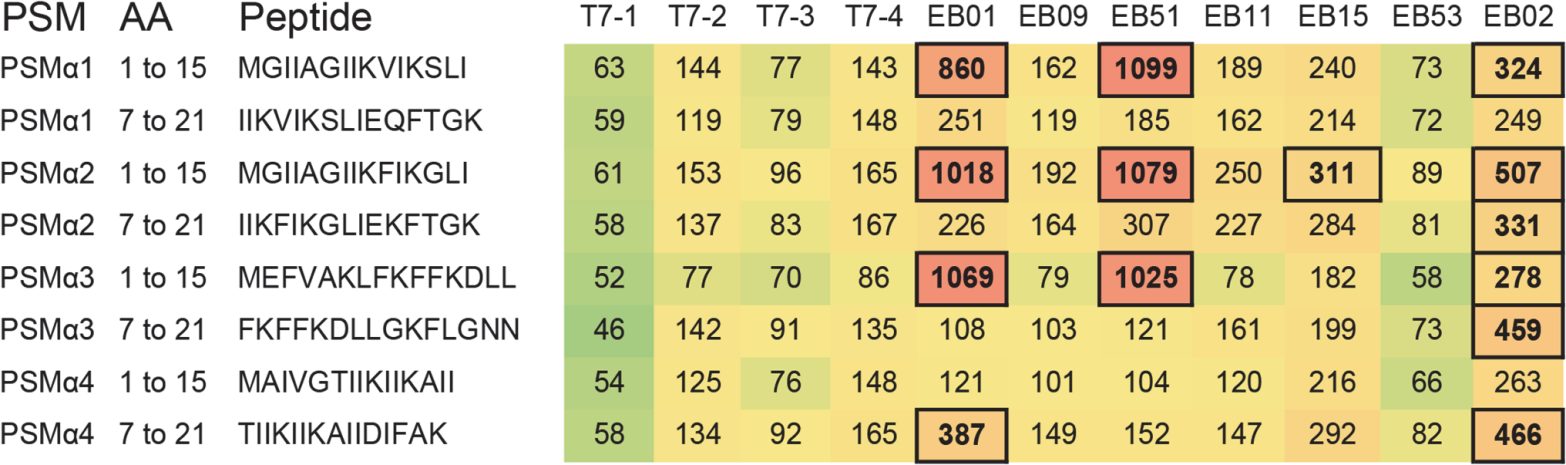
Heatmap of anti-PSMα1-4 reactivity. Plot of IgG responses from sera of volunteers (T7-1 till T7-4) and EB patients (EB01, 09, 51, 11, 15, 53, 02) against the N- (1 to 15) and C- (7 to 21) terminal parts of the PSMα1-4 peptides. Colors represent a gradient of reactivity against the various peptides (green is low and red is high). Peptides with a signal exceeding the median plus twice the standard deviation are boxed.

As the PSMα1 has previously been shown to be exposed on the cell surface of *S*. *aureus*, we included the PSMα1, α2, α3, and α4 in an epitope mapping approach using the PepScan technology [38]. This analysis showed that 5 out of 7 EB patients have an above average titer of IgGs against the peptides tested (Fig. 3). Three out of 7 plasma samples from EB patients showed a very high reactivity against the N-terminal regions of the PSMα1, α2, and α3 peptides. These results show that besides the IsaA-His_6_, LytM-His_6_ and Nuc-His_6_ proteins, the PSM peptides are also well recognized by the humoral immune system.

### Immunization with an octa-valent mixture of *S*. *aureus* antigens does not reduce bacterial load in mice with *S*. *aureus* isolate P bacteremia

**Fig 4:**
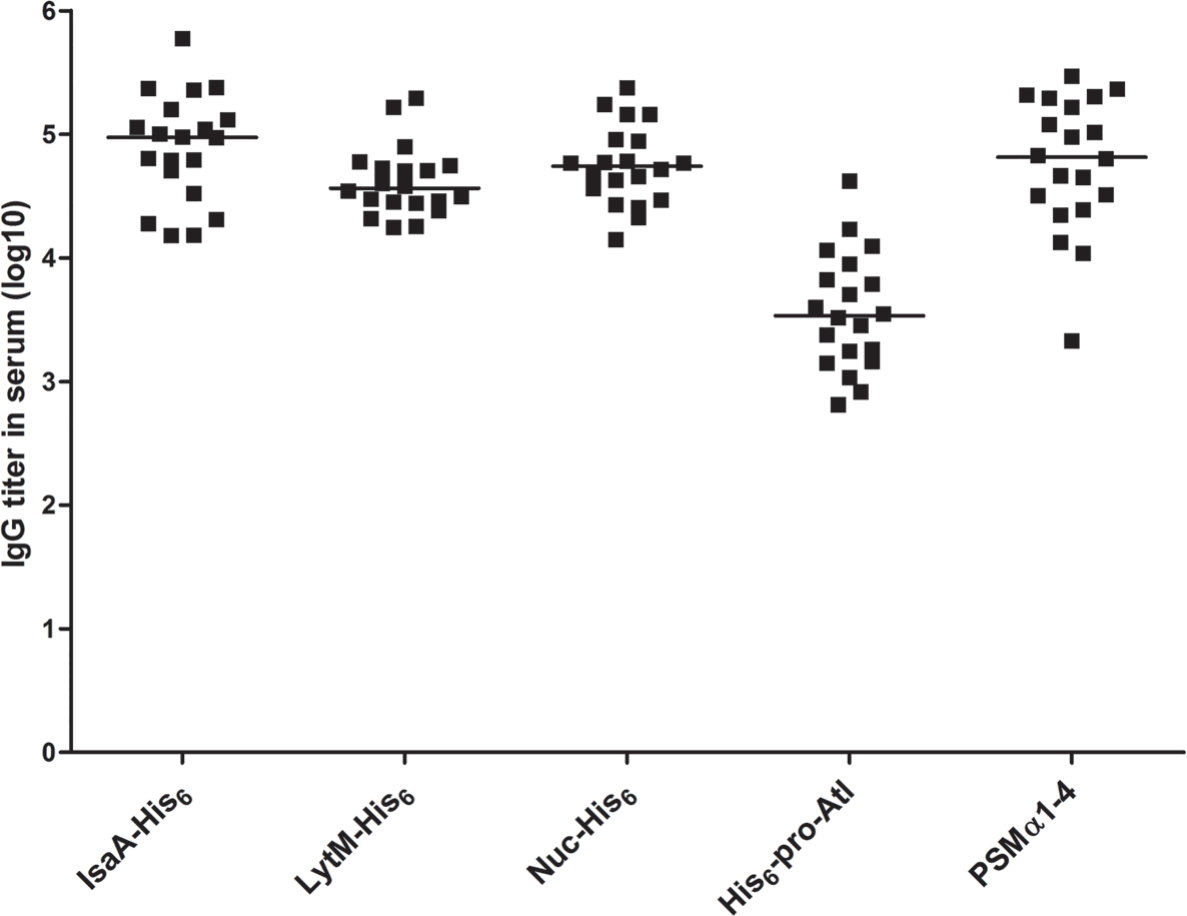
IgG titers in serum of mice after immunization with the octa-valent mixture. Mice (n = 20) were immunized subcutaneously with the mixture containing IsaA-His_6_, LytM-His_6_, Nuc-His_6_, His_6_-pro-Atl, and PSMα1-4 at days -70 (5 μg of each antigen), days -42, and -14 (25 μg of each antigen). IgG titers **o**n day -1 were assessed. Each symbol represents a single mouse. Median values are indicated by horizontal lines.

Groups of mice were immunized by subcutaneous injection with 5 μg of purified IsaA-His_6_, LytM-His_6_, Nuc-His_6_, His_6_-pro-Atl, and PSMα1-4 each, emulsified in TiterMax Gold adjuvant on day -70. On days -42 and -14, they were boosted with this mixture containing 25 μg of each purified antigen. Blood samples were collected before and after immunization, and specific serum IgG levels were determined by ELISA, demonstrating that these antigens generated humoral immune responses to immunization (Fig. 4). The response against His_6_-pro-Atl was on average 10-fold lower compared to the other 4 antigens.

**Fig 5:**
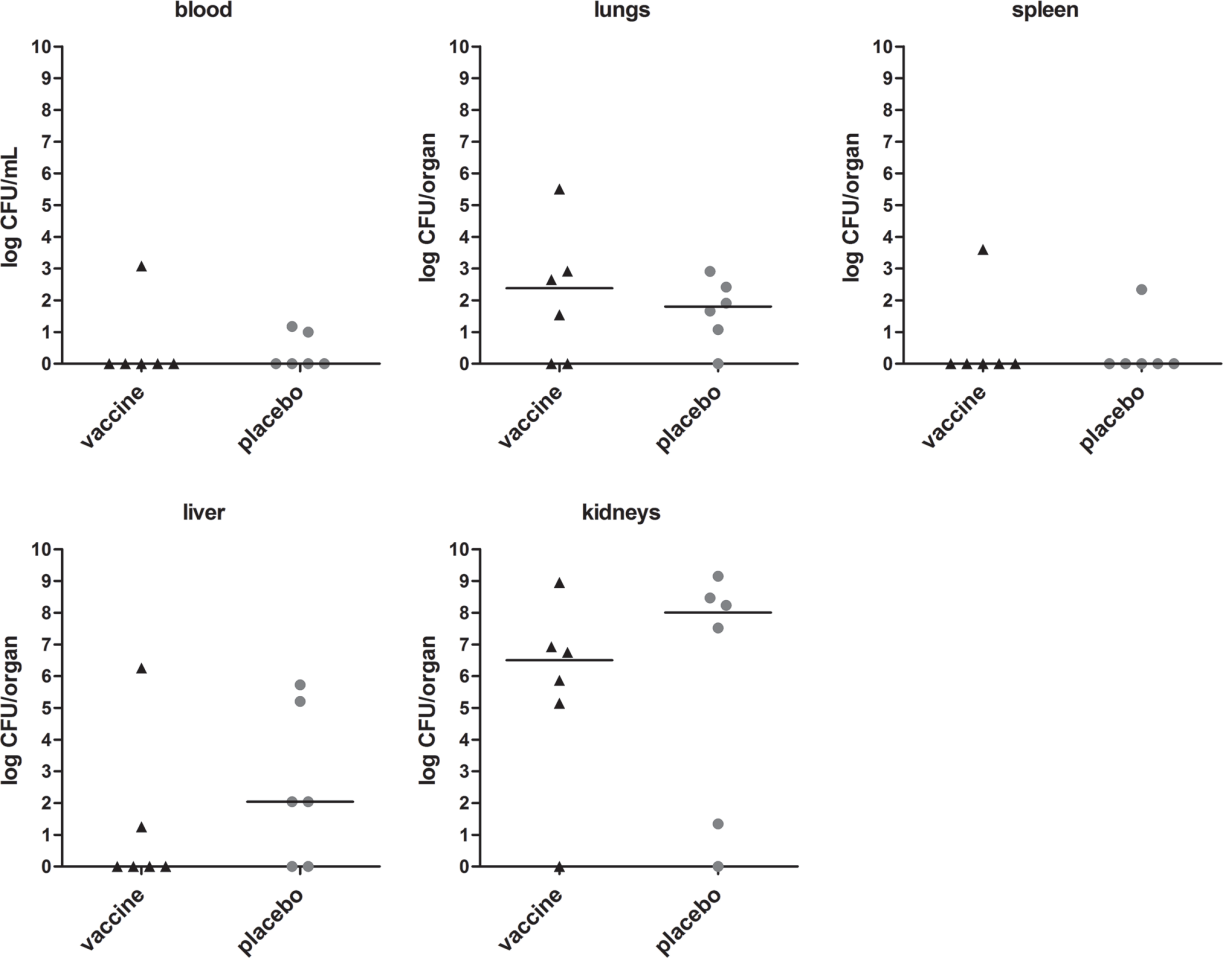
Bacterial load in immunized mice with *S*. *aureus* isolate P bacteremia. Mice (n = 8) were immunized with the octa-valent vaccine containing IsaA-His_6_, LytM-His_6_, Nuc-His_6_, His_6_-pro-Atl, and PSMα1-4, or with placebo. Animals were infected by intravenous inoculation of *S*. *aureus* isolate P (3 × 10^5^ CFU). At day 14, surviving mice were sacrificed and quantitative cultures of blood, lungs, spleen, liver, and kidneys were performed. Each symbol represents a single mouse. Median values are indicated by horizontal lines. Statistically significant differences in *S*. *aureus* load were not observed (*P* > 0.05; Mann-Whitney U test).

Mice were challenged at day 0 with mild *S*. *aureus* isolate P bacteremia (n = 8 immunized mice, n = 8 placebo-immunized mice), and animal discomfort and survival rate over 14 days after infection were monitored. At day 14, surviving mice were sacrificed, and blood and organs were removed for assessment of the bacterial load. In placebo-immunized mice, discomfort increased, while animal survival declined over time. At day 14, 6 out of 8 placebo-immunized mice were still alive. *S*. *aureus* load in kidneys of these mice was high, while bacterial load in lungs and liver was low, and blood and spleen were culture negative (Fig. 5).

Compared to placebo-immunized animals, immunization with the octa-valent antigen mixture did not reduce the *S*. *aureus* load in blood, lungs, spleen, liver, and kidneys (Fig. 5). Furthermore, discomfort score and animal survival rate over 14 days did not differ between immunized and placebo-immunized mice (data not shown).

### Immunization with an octa-valent *S*. *aureus* antigen mixture is not protective against mortality due to *S*. *aureus* USA300 bacteremia in mice

**Fig 6:**
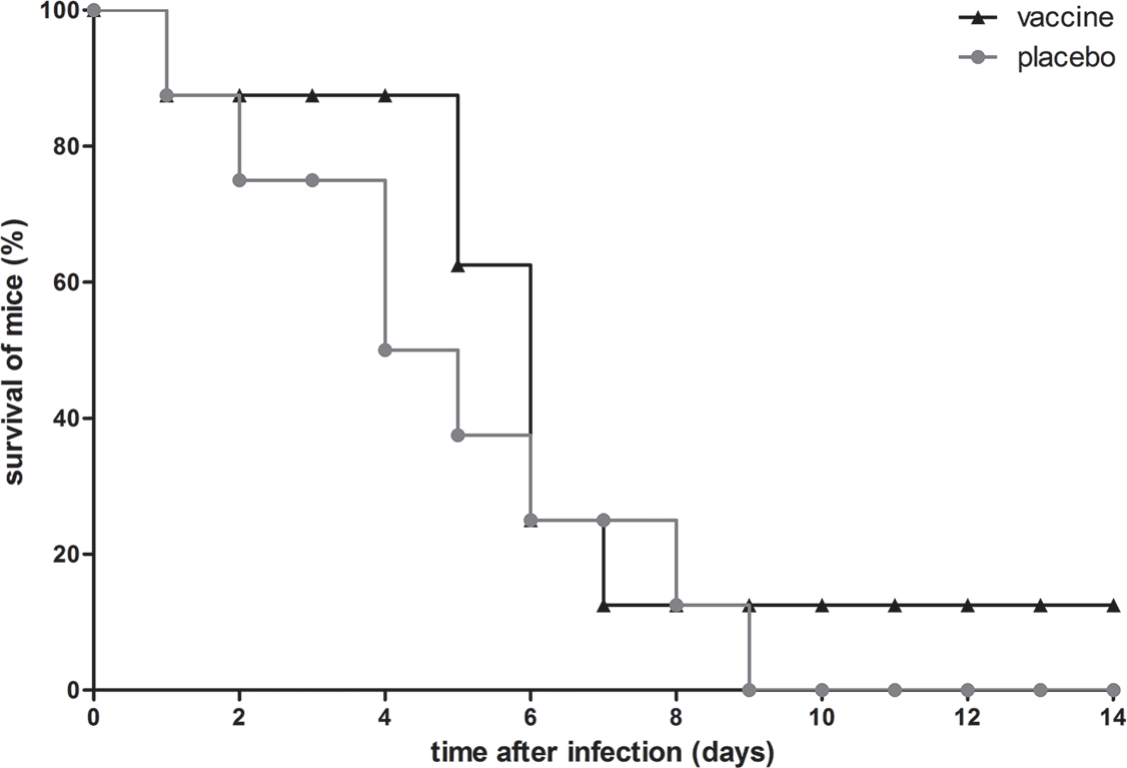
Survival of immunized mice with *S*. *aureus* USA300 bacteremia. Mice (n = 8) were immunized with the octa-valent vaccine containing IsaA-His_6_, LytM-His_6_, Nuc-His_6_, His_6_-pro-Atl, and PSMα1-4, or with placebo. Animals were infected by intravenous inoculation of *S*. *aureus* USA300 (6 × 10^5^ CFU), and were monitored for 14 days. A statistically significant difference in animal survival rates was not observed (*P* > 0.05; log rank test).

To assess whether the lack of protective effect of immunization with the octa-valent mixture was *S*. *aureus* strain-dependent, we included a lethal mouse model of MRSA bacteremia as well. As immunized mice showed excellent IgG titers, the immunization schedule was not adapted. In this model, mice were challenged with severe *S*. *aureus* USA300 bacteremia (n = 8 immunized mice, n = 8 placebo-immunized mice), and animal discomfort and survival rate over 14 days after infection were monitored. In placebo-immunized mice, discomfort increased, while animal survival declined gradually over time, and at day 9, all placebo-immunized mice had died (Fig. 6).

In this model of severe MRSA bacteremia, immunization with the octa-valent mixture containing IsaA-His_6_, LytM-His_6_, Nuc-His_6_, His_6_-pro-Atl, and PSMα1-4 did not protect against mortality due *S*. *aureus* USA300 bacteremia (Fig. 6). Furthermore, the discomfort score over 14 days was not reduced, and higher IgG levels did not correlate with increased time-to-death of mice (data not shown).

### Immunization with an octa-valent *S*. *aureus* antigen mixture does not protect against S. aureus isolate P skin infection

**Fig 7:**
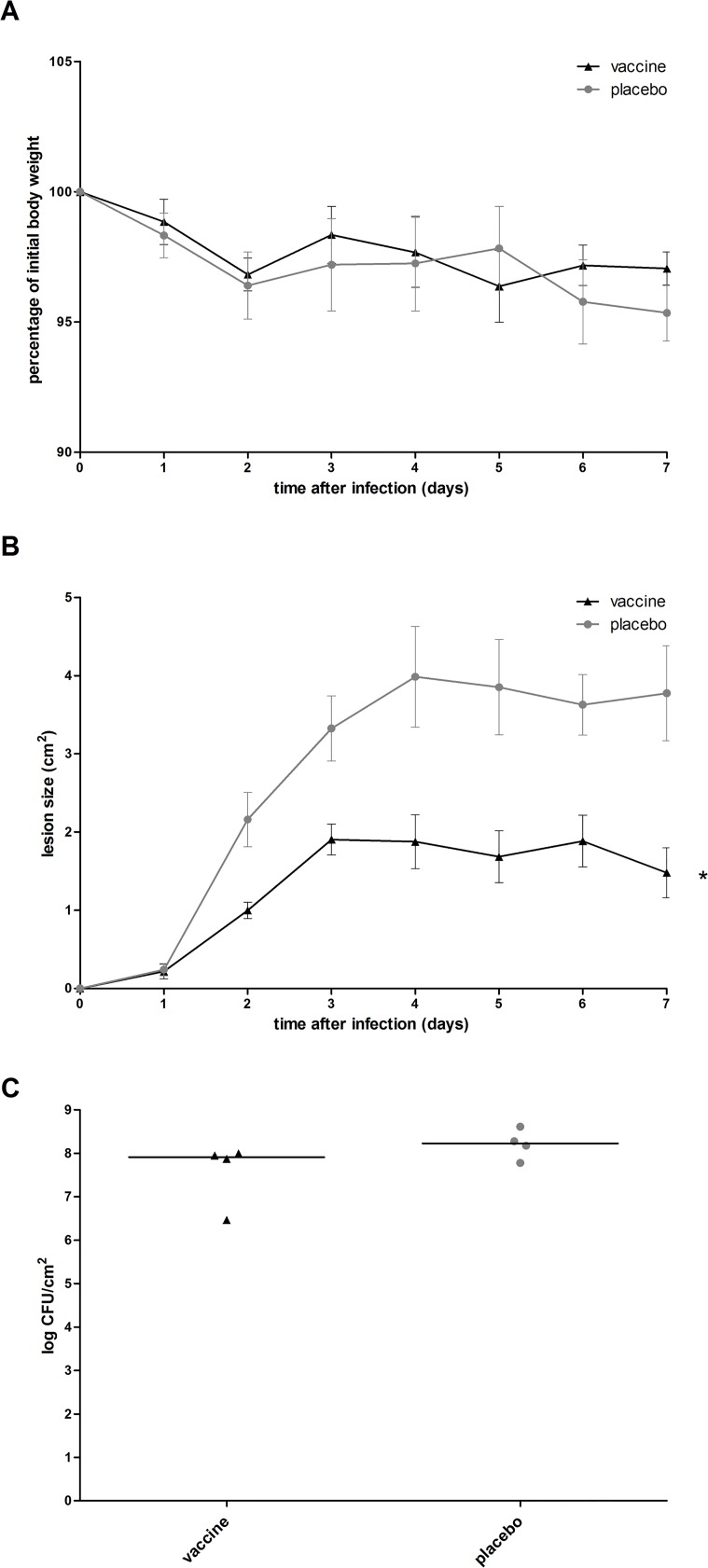
Course of infection in immunized mice with *S*. *aureus* isolate P skin infection. Mice (n = 4) were immunized with the octa-valent vaccine containing IsaA-His_6_, LytM-His_6_, Nuc-His_6_, His_6_-pro-Atl, and PSMα1-4, or with placebo. Animals were infected by intradermal inoculation of *S*. *aureus* isolate P (3 × 10^7^ CFU), and were monitored for 7 days. (A) Animal body weight over time after *S*. *aureus* skin infection was not affected by vaccination (*P* > 0.05; Quade’s rank analysis of covariance). (B) Lesion size was significantly reduced by vaccination, as indicated by the asterisk (*P* = 0.005; Quade’s rank analysis of covariance). (C) *S*. *aureus* load in the skin lesion at day 7 was not reduced by vaccination (*P* > 0.05; Mann-Whitney U test).

Absence of protection of the octa-valent mixture in *S*. *aureus* bacteremia does not exclude a possible (lack of) protection in other types of *S*. *aureus* infections. To assess whether this antigen mixture elicits a protective immune response against *S*. *aureus* skin infection, immunized mice were challenged with *S*. *aureus* isolate P via the intradermal route (n = 4 immunized mice, n = 4 placebo-immunized mice). Animal body weight and lesion size over 7 days after infection were monitored. At day 7, mice were sacrificed and the *S*. *aureus* load in skin lesions was assessed. In placebo-immunized mice, body weight loss over 7 days was minor, with a maximum of 6.5%. Size of the skin lesion increased over time until 2.8–5.2 cm^2^ at day 7. At this time point, median *S*. *aureus* load in the skin lesion was 2 × 10^8^ CFU/cm^2^ (Fig. 7).

Compared to placebo-immunized mice, immunization with the octa-valent antigen mixture did not limit the body weight loss over time (Fig. 7A), while size of the skin lesion was reduced in immunized mice (Fig. 7B). *S*. *aureus* load in skin lesions at day 7 was not reduced in immunized mice (Fig. 7C). As reduction of the bacterial load in the skin lesion was our primary outcome measure, we concluded that immunization with the octa-valent *S*. *aureus* antigen mixture did not protect against *S*. *aureus* isolate P skin infection.

## Discussion

In view of the high mortality rates of *S*. *aureus* infections [42–45], the emergence of antibiotic-resistant *S*. *aureus* strains [6] and the lack of new antimicrobials in the development pipeline [8], alternative treatment strategies for *S*. *aureus* infections are urgently needed. One approach is treatment through immunization targeting *S*. *aureus* antigens. This may be an interesting substitute for or additive to the currently used antibiotics. A role of certain anti-staphylococcal antibodies in protection against *S*. *aureus* infection-related death is suggested by a number of studies in humans [14,26,46,47]. Next to these suggestions from clinical studies, a number of studies in *S*. *aureus*-infected experimental animals showed protective effects of active or passive immunization. Notwithstanding these promising results obtained in experimental animals, no efficacy of immunization is observed in clinical studies [9–11].

The lack of protective capacity of anti-staphylococcal immunization may be explained by, for example, a lack of power of several clinical studies [12,13], the limited number of *S*. *aureus* antigens targeted in these studies (mostly one or two), or the *S*. *aureus* infection studied (mostly bacteremia/sepsis). In addition, the apparent lack of success of immunization targeting for example CP5 and CP8 [48,49], IsdB [50], or ClfA [51] in *S*. *aureus*-infected patients may be related to the choice of targets for immunization. Therefore, in the present study, we selected *S*. *aureus* antigens that are accessible for antibodies based on proteomic analysis of exoproteomes of *S*. *aureus* [15] and a proteolytic shaving approach of *S*. *aureus* [17]. This strategy resulted in the selection of IsaA, LytM, Nuc, pro-Atl, and PSMα1-4 as targets for active immunization. Previous studies showed that these eight *S*. *aureus* antigens are immunogenic in humans [14,26,29,30], and may therefore be applicable in the clinical setting. Moreover, monoclonal antibodies against IsaA enhance the killing of *S*. *aureus* in whole blood samples from healthy subjects and patients prone to staphylococcal infections [52], and passive immunization with these monoclonal antibodies can lead to protection against *S*. *aureus* infections in mice [28,53].

Using the hosts *L*. *lactis* and *E*. *coli*, all selected *S*. *aureus* antigens (IsaA, LytM, Nuc, and pro-Atl) were successfully isolated. No major degradation products were observed after isolation, and IsaA-His_6_, LytM-His_6_, and Nuc-His_6_ fusion proteins were all biologically active after purification, indicating they had retained their natural conformation. In addition, these three fusion proteins as well as the PSMα1-4 peptides were well recognized by IgG present in plasma of healthy volunteers and EB patients. These results indicate that His-tagged fusions did not affect binding of IgG to these antigens. In addition, in a previous study, it has been shown that the Atl propeptide is recognized by antibodies present in sera of EB patients (our unpublished data).

The potentially protective capacity of immunization was evaluated in mice immunized with a mixture of IsaA, LytM, Nuc, pro-Atl and PSMα1-4. In the use of an octa-valent antigen cocktail we followed the approach of Stranger-Jones et al. [54], who immunized mice with IsdA, IsdB, SdrD, and SdrE, either in a combination or individually. They observed that single antigen immunization elicited no or only very modest protection against *S*. *aureus* abscess formation or *S*. *aureus* lethal challenge, whereas immunization with the cocktail completely protected in both *S*. *aureus* infections. In addition, Bagnoli et al. proposed a model in which vaccine efficacy is gained via antibodies that directly inhibit bacterial viability and/or toxicity, via antibodies to mediate opsonophagocytosis, and via cell-mediated immunity to stimulate recruitment of phagocytes at the site of infection [55]. For this purpose, multiple *S*. *aureus* antigens with different functions should be targeted. In the present study immunization of mice with the octa-valent antigen mixture of IsaA, LytM, Nuc, pro-Atl and PSMα1-4 resulted in detectable IgG responses against all eight antigens. Although anti-pro-Atl levels were slightly lower, all observed IgG levels were in the expected range [54,56]. This observation indicated that all antigens were recognized well by the immune system of the mice.

After the final booster immunization, mice were infected with *S*. *aureus*. Clinically relevant models were used: mild bacteremia by *S*. *aureus* isolate P (MSSA), lethal bacteremia by *S*. *aureus* USA300 (MRSA), and transient *S*. *aureus* isolate P skin infection. It was shown that active immunization with the octa-valent mixture of IsaA-His_6_, LytM-His_6_, Nuc-His_6_, His_6_-pro-Atl, and PSMα1-4, although resulting in high anti-staphylococcal IgG levels, did not protect mice against mild *S*. *aureus* isolate P bacteremia, severe *S*. *aureus* USA300 bacteremia, or *S*. *aureus* isolate P skin infection.

The lack of protection by active immunization with the octa-valent *S*. *aureus* antigen mixture in our mouse infection experiments may be related to the study design or the presumed role of anti-staphylococcal antibodies in protection against these *S*. *aureus* infections. Regarding the study design, the *S*. *aureus* isolates used and the type of *S*. *aureus* infections studied are clinically relevant [15,31,32]. Other investigators used these infection models as well, and demonstrated that active or passive immunization targeting *S*. *aureus* antigens can lead to protection in mice [9–11]. In addition, both *S*. *aureus* strains produced all eight *S*. *aureus* antigens. It should be noted that preclinical animal models of *S*. *aureus* infection do not fully mimic the natural *S*. *aureus* infection process in humans due to various differences among which differences in host cell proteins such as hemoglobin [57] and the requirement for high bacterial inocula [58] in the non-human host. Despite these limitations of experimental animal models, we conclude that failure to show protective activity upon active immunization can presumably not be explained by the choice of *S*. *aureus* strains or the choice of infection models. As the observed differences between immunized mice and placebo-immunized mice were not significant, not even borderline, increasing the group sizes will probably not result in significant differences. Therefore, this cannot clarify the failure of immunization in our *in vivo* models.

A more plausible explanation for the lack of protection of the octa-valent antigen mixture in the present study may be related to the presumed role of anti-staphylococcal antibodies in protection against *S*. *aureus* infection. Although previous studies suggested a role of anti-staphylococcal antibodies in protection against death in *S*. *aureus* carriers [14,26,46,47,56,59], their role may be overestimated. Possibly, IgG against IsaA-His_6_, LytM-His_6_, Nuc-His_6_, His_6_-pro-Atl, and PSMα1-4, or anti-staphylococcal IgG in general, has no or only a limited role in protection against *S*. *aureus* infections. It may be conceivable that antibodies against *S*. *aureus* antigens in humans are just a result of prior exposure in carriers and non-carriers, via *S*. *aureus* colonization or previous (sub)clinical *S*. *aureus* infection, while their protective capacity is limited. Previous exposure to *S*. *aureus* in carriers may also result in improved cellular immunity, which could also protect against *S*. *aureus* infection-related death, as was already suggested by Joshi et al. [60]. Beside this, the production of virulence factors such as toxins and immune evasion proteins by *S*. *aureus* [61,62] might overwhelm the generated humoral immune response. Another reason for the failure of the octa-valent antigen mixture to protect mice against *S*. *aureus* infection might be related to a potential immunosuppression induced by one of the antigens in the cocktail. As other combinations of *S*. *aureus* antigens were not tested, conclusions in this respect cannot be drawn.

Although passive immunization with monoclonal antibodies against IsaA protected mice against *S*. *aureus* infection [28,53], in the present study no protection was obtained after active immunization with a mixture including IsaA. A possible explanation for this discrepancy may be related to insufficient binding of polyclonal antibodies induced by active immunization to relevant epitopes of IsaA in order to provide protection against *S*. *aureus* infection, in contrast to the monoclonal antibodies administered by passive immunization, clearly binding to relevant epitopes of IsaA.

In conclusion, active immunization with an octa-valent mixture containing IsaA-His_6_, LytM-His_6_, Nuc-His_6_, His_6_-pro-Atl and the PMSα1–4 peptides does not protect mice against *S*. *aureus* bacteremia and *S*. *aureus* skin infection. The observations suggest that these polyclonal anti-staphylococcal antibodies do not provide protection against *S*. *aureus* infection. Consequence of the present study should not be abandoning of research focusing on immunization in *S*. *aureus* infections, as other investigators obtained promising results in this respect. Instead, future research should focus on novel treatment strategies combining immunization with antibiotic treatment and/or cytokine administration.
